# Molecular Dysfunctions of Mitochondria-Associated Membranes (MAMs) in Alzheimer’s Disease

**DOI:** 10.3390/ijms21249521

**Published:** 2020-12-14

**Authors:** Fanny Eysert, Paula Fernanda Kinoshita, Arnaud Mary, Loan Vaillant-Beuchot, Frédéric Checler, Mounia Chami

**Affiliations:** 1 Laboratory of Excellence DistAlz, INSERM, CNRS, Institut of Molecular and Cellular Pharmacology, Université Côte d’Azur, Sophia-Antipolis, 06560 Valbonne, France; eysert@ipmc.cnrs.fr (F.E.); kinoshita@ipmc.cnrs.fr (P.F.K.); mary@ipmc.cnrs.fr (A.M.); vaillant@ipmc.cnrs.fr (L.V.-B.); checler@ipmc.cnrs.fr (F.C.); 2 Instituto de Ciências Biomédicas, Department of Pharmacology, Universidade de São Paulo, São Paulo 05508-900, Brazil

**Keywords:** endoplasmic reticulum (ER), mitochondria, mitochondria-associated membranes (MAMs), Alzheimer’s disease (AD), calcium signaling, phospholipids, cholesterol, fatty acid, lipids, unfolded protein response (UPR), APOE, tau, β amyloid precursor protein (APP), amyloid β peptide (Aβ), APP-C-terminal fragments (APP-CTFs)

## Abstract

Alzheimer’s disease (AD) is a multifactorial neurodegenerative pathology characterized by a progressive decline of cognitive functions. Alteration of various signaling cascades affecting distinct subcellular compartment functions and their communication likely contribute to AD progression. Among others, the alteration of the physical association between the endoplasmic reticulum (ER) and mitochondria, also referred as mitochondria-associated membranes (MAMs), impacts various cellular housekeeping functions such as phospholipids-, glucose-, cholesterol-, and fatty-acid-metabolism, as well as calcium signaling, which are all altered in AD. Our review describes the physical and functional proteome crosstalk between the ER and mitochondria and highlights the contribution of distinct molecular components of MAMs to mitochondrial and ER dysfunctions in AD progression. We also discuss potential strategies targeting MAMs to improve mitochondria and ER functions in AD.

## 1. Introduction

Alzheimer’s disease (AD) is a neurodegenerative disease characterized by two major histological hallmarks: (1) the neurofibrillary tangles (NFTs) corresponding to intracellular aggregates of abnormally hyperphosphorylated Tau protein (pTau) and, (2) senile plaques that are mainly composed of extracellular aggregates of β amyloid peptide (Aβ) derived from the sequential cleavage of its precursor, the amyloid precursor protein (APP), by β-secretase and γ-secretase enzymes [1,2]. Late-onset AD (LOAD) cases are considered to be sporadic (SAD) accounting for the majority of AD cases. For a long time, apolipoprotein E (*APOE*) has been described as the sole and main genetic risk factor for LOAD forms [3]. Recently, a genome wide-association study (GWAS) identified several new AD genetic risk factors [4,5,6]. Dominantly inherited familial AD (FAD) accounts for less than 1% of the cases and can be caused by mutations in *APP*, *presenilin 1* (*PS1*), or *presenilin 2 (PS2)* genes [7,8]. Overall, AD is now considered a complex pathology, the mechanistic defects of which remain unclear. The amyloid cascade is the most widely accepted hypothesis in AD and proposes Aβ accumulation as the etiological trigger of the pathology. This has been supported by genetic evidence demonstrating that FAD mutations in genes coding for APP or its processing enzymes (PS1 and PS2) constituting the catalytic components of the γ-secretase enzymatic complex [9] lead to increased production and secretion of Aβ peptides [9]. The latter accumulate in the form of neurotoxic Aβ oligomers (Aβo) and are thought to trigger several stress responses in neurons leading to the onset of neurofibrillary degeneration [9]. Several therapeutic approaches were developed with the aim to reduce the accumulation of Aβ through active and passive immunizations. However, the failure of these trials to rescue cognitive declines or even stabilize them [10] casts some doubts about the amyloid cascade hypothesis. Notably, the sole contribution of Aβ to AD pathogenesis is challenged, since APP processing yields several fragments besides Aβ [2]. In physiological conditions, 90% of mature APP is cleaved by α-secretase at the plasma membrane, producing the secreted soluble α-APP (sAPPα) and the membrane-anchored APP-C-terminal (APP-CTF)α fragment (C83). The latter is further cleaved by γ-secretase producing p3 and AICD (APP intracellular domain) peptides. On the other hand, 10% of mature APP is cleaved by β-secretase (BACE1) following its internalization towards the endosomal/lysosomal pathway, producing the soluble β-APP (sAPPβ) released in the extracellular environment, and the membrane-anchored APP-CTFβ fragment (C99), which is further cleaved by γ-secretase producing AICD and Aβ peptides [2]. C99 is also cleaved by α-secretase to produce C83 [11]. Furthermore, other APP-derived fragments produced by other enzymes (such as η- and δ- secretases) were recently described [12,13,14], likely contributing to AD pathophysiology [12,13,14,15,16,17,18].

**Figure 1:**
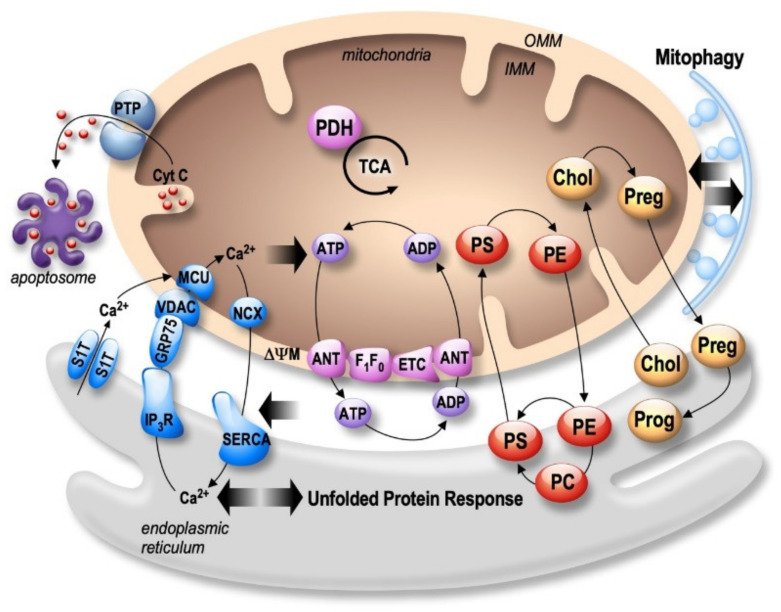
Bidirectional regulation of ER and mitochondria functions in MAMs. Mitochondria Ca^2+^ uptake (i.e., by the VDAC (voltage-dependent anion channel) located in the outer mitochondria membrane (OMM) and the mitochondrial Ca^2+^ uniporter complex (MCU) located in the inner mitochondria membrane (IMM)) increases the activity of mitochondrial enzymes (i.e., the pyruvate dehydrogenase (PDH), and the tricarboxylic acid cycle (TCA)) orchestrating the activity of the oxidative phosphorylation electron transport chain (ETC) producing ATP by the F1F0 ATP-synthase. ATP is then transported through the adenine nucleotide translocator (ANT). In return, ATP is used by the sarco-endoplasmic reticulum Ca^2+^ ATPase (SERCA) ensuring active Ca^2+^ storage in the ER, necessary for ER functions (i.e., protein synthesis and maturation). Mitochondrial Ca^2+^ homeostasis results from an equilibrium between Ca^2+^ uptake and extrusion by the Na^2+^/Ca^2+^ exchanger (NCX). Prolonged or excessive mitochondrial Ca²^+^ uptake triggers mitochondrial membrane permeability transition pore (PTP) opening, initiating cytochrome c (Cyt c) release, apoptosome formation, and the activation of apoptotic cell death. In particular, altered ER Ca^2+^ homeostasis is associated with the unfolded protein response (UPR), triggering excessive transfer of Ca^2+^ from the ER to mitochondria (i.e., through SERCA1 truncated isoform (S1T), or the inositol 1,4,5-trisphosphate receptors (IP_3_R)), which is tightly linked to ER stress-mediated mitochondria apoptotic cell death. In parallel, MAMs control phospholipids biosynthesis and transfer between organelles where phosphatidylcholine (PC); phosphatidylethanolamine (PE); and phosphatidylserine (PS) are the most abundant ones. MAMs are also the intersection site of transport and metabolism of cholesterol (Chol), which is catalyzed to generate pregnenolone (Preg) and released to the ER to produce progesterone (Prog). MAMs-associated lipid microdomains regulate the autophagic/mitophagic processes through the recruitment of autophagic proteins and autophagosome formation.

Both the endoplasmic reticulum (ER) and mitochondria are vital organelles of the cell. Rough and smooth ER participate in protein synthesis, folding, and transport. The ER is also involved in other fundamental cellular functions such as lipids- and carbohydrates- metabolism and is the major calcium (Ca²^+^) storage pool [19]. On the other hand, mitochondria act as the powerhouse of the cell by generating energy through adenosine triphosphate (ATP) production [20]. Besides, mitochondria play a major role in buffering the Ca²^+^ flux from the ER, but also in lipid- and amino acid- metabolism, beneficial and harmful reactive oxygen species (ROS) production, and apoptosis [20]. It is now well established that ER and mitochondria functions are highly interconnected. They physically interact to form specific microdomains called mitochondria-associated ER membranes (MAMs), where the outer mitochondrial membrane (OMM) is close to the ER in the order of 10–100 nanometers [21,22,23]. The maintenance of stable contact sites between the ER and mitochondria provides a platform for bidirectional crosstalk. Not surprisingly, MAMs control intracellular elementary events such as Ca²^+^ homeostasis, metabolic flow, protein transport [24], mitochondrial lipids production, phospholipid biosynthesis, mitochondrial fusion and fission, and global events such as autophagy and apoptosis [25] (Figure 1). In addition, MAMs are a hot-spot for the transfer of stress signals from the ER to mitochondria, particularly under ER stress conditions activating the unfolded protein response (UPR) [26] (Figure 1). As a result, one can argue that the alteration of ER and mitochondria communication may influence and disrupt these functions, thus leading to the development of several pathologies and vice-versa.

**Figure 2:**
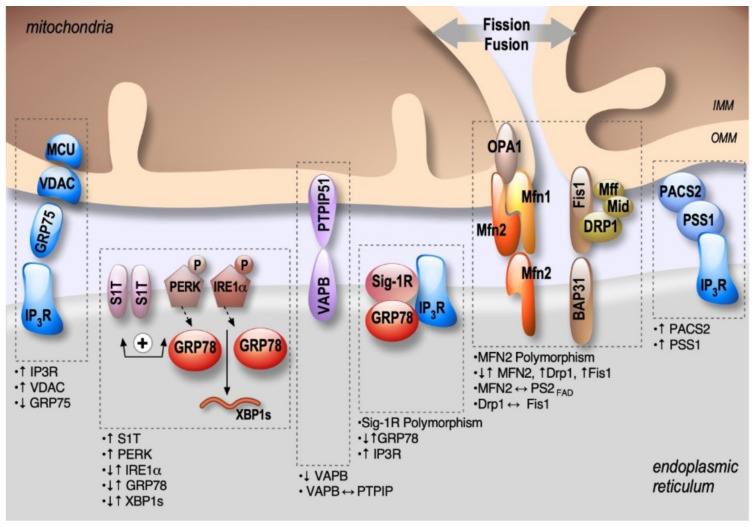
Schema showing the localization and alterations of MAMs molecular components in AD, including upregulation (↑) or down-regulation (↓) of MAMs proteins expression, MAMs proteins interactions (⟷), and genes polymorphism.

**Figure 3:**
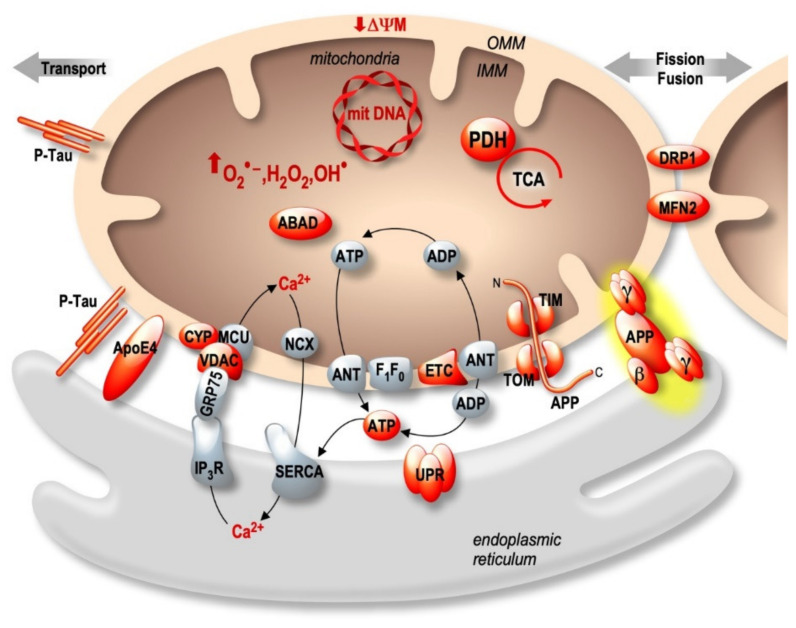
APP and its derived fragments APP-CTFs and Aβ; presenilins (PSs), pTau and APOE4 are involved in the impairment of the expression and activity of several mitochondrial proteins (highlighted in red) and interfere with UPR in the ER. APP localizes and is processed by β- and γ-secretases in MAMs. AD Molecular partners also disturb MAMs structure and function.

ER stress and mitochondrial dysfunctions occur early in AD, likely contributing to disease progression and irreversible neuronal death [27,28,29,30]. Numerous papers reported that several pathogenic paradigms associated with AD are closely linked to MAMs structure and function alterations. This review aims at delineating the contribution of several structural and functional partners of MAMs (i.e., Mitofusins (MFNs), Protein Kinase-like Endoplasmic Reticulum Kinase (PERK), the serine/threonine-protein kinase/endoribonuclease inositol-requiring enzyme 1α (IRE1α), and the sarco/endoplasmic reticulum Ca^2+^ ATPase 1 (SERCA) truncated isoform (S1T)), and MAMs complexes (i.e., IP_3_R-Grp75-VDAC, PACS2-PSS1, BAP31-FIS1-DRP1, VAPB-PTPIP51, and Sig-1R-Grp78) (Figure 2) in pathophysiological processes of AD. We also elucidate how AD associated proteins (APP and its derived fragments, PSs, Tau, and APOE) may impact MAMs structure and function (Figure 3). Finally, we highlight the potential relevance of MAMs molecular components as therapeutic targets to prevent the development of AD.

## 2. Structural and Functional Partners of MAMs Are Involved in AD

### 2.1. Mitofusins: MFNs

Mitofusins (MFN1 and MFN2) belongs to GTPases family and are located in the OMM. Both MFNs are involved in mitochondrial fusion process by forming homotypic and heterotypic interactions with the inner mitochondria membrane’s (IMM) OPA1 protein [31,32]. Besides, several studies proposed that MFN2 modulates mitochondrial membrane potential (ΔΨm) through the regulation of nuclear-encoded subunits of OXPHOS complexes I, II, III, and V [33]. MFN2 acts as a regulator of apoptosis in a cell type- and age-specific manner [34,35], and is linked to mitophagy [36]. The importance of MFN2 in organelles juxtaposition and the communication function of MAMs has been also proven through the regulation of mitochondria Ca^2+^ uptake [37]. MFN2 was also shown to be involved in dendritic outgrowth, spine formation in mice models, and to prevent neurodegeneration in the cerebellum through fusion mechanism [38].

Several studies reported that MFN2 is associated with AD [39] (Figure 2 and Table 1). MFN2 gene polymorphisms were reported in LOAD [40]. Moreover, the mitochondrial fusion and fission balance is altered in AD, correlating with enhanced or reduced MFNs expression [41]. A reduction of both MFN1 and MFN2 expression was reported in human AD brains [41,42]. Accordingly, a reduction of MFN2 expression was also reported in N2a cells expressing the APP familial Swedish mutation (APPswe) that overproduces Aβo [43]. On the contrary, an overexpression of MFN2 was observed in human cybrid cells in which mitochondria from mild cognitive impairment (MCI) subjects were incorporated into neuronal cells depleted of endogenous mitochondrial DNA [44]. These discrapencies may point out a complex regulation of MFN2 in AD pathogenesis. In fact, directly linked to APP processing in AD, MFN2 was shown to control γ-secretase activity and Aβ production by modulating ER and mitochondrial membrane contacts, without affecting BACE1 and neprilysin expression [45]. Moreover, PS2-linked FAD mutants promote ER-mitochondria coupling in a MFN2-dependent manner (Table 1) [46]. Altogether, these studies highlight a central role of MFN2 in AD pathogenesis through the regulation of MAMs tethering, mitochondrial structure and function, and cell death but also APP processing.

### 2.2. IP_3_R-Grp75-VDAC

The inositol 1,4,5-trisphosphate receptors (IP_3_Rs), releasing Ca^2+^ from the ER to the cytosol [47], are enriched in ER-mitochondria contact sites playing a key role in cellular differentiation, survival, and apoptosis [48,49]. Ca^2+^ uptake by mitochondria occurs through VDAC, an OMM protein which is a permeable Ca^2+^ channel, together with the mitochondrial Ca^2+^ uniporter (MCU) complex [50] located on the IMM (Figure 1). In MAMs, a functional complex between VDAC (isoform 1) and IP_3_R (isoform 3) is formed through a chaperone called glucose-regulated protein 75 (Grp75, a member of the heat shock protein 70 family) [51]. The IP_3_R-Grp75-VDAC complex regulates Ca²^+^ transfer from the ER to mitochondria, suggesting its involvement in Ca²^+^ homeostasis deregulation largely described in AD [51]. While there is only one study documenting a reduction of Grp75 expression in the temporal and parietal cortex of AD post-mortem brains [52], several studies demonstrated alterations of VDAC expression in AD (Figure 2 and Table 1). VDAC1 expression increases progressively according to AD stages in the cortex of post-mortem brains, in aged APP mice, and also in neuroblastoma cells treated with Aβo [53]. Accordingly, another study reported that Aβ treatment of primary hippocampal neurons increased VDAC1 and IP_3_R expression, resulting in enhanced contacts between ER and mitochondria [54]. VDAC1 also interacts with Aβ and pTau, likely contributing to mitochondrial dysfunction [55]. In turn, Aβ triggers VDAC dephosphorylation in lipid rafts in AD brains correlating with cell death [56]. Inversely, reduced expression of VDAC1 decreased the mRNA levels of APP, Tau, PS1 and PS2, and also BACE1 [57].

Altearations of IP_3_Rs expressions and activity have been largely explored in AD and were demonstrated to be linked to both Aβ and PS1 and PS2 FAD mutations. Fibroblasts from FAD patients exhibited an enhanced response to bombesin- and bradykinin-mediated IP3 generation in comparison to control cells [58]. These data were further confirmed in cortical and hippocampal neurons from 3xTgAD mice [59]. Aβo are also involved in IP_3_R overactivation by increasing IP_3_ production and triggering cytotoxicity [60]. Importantly, the reduction of IP_3_R1-mediated Ca^2+^ release in the cortical and hippocampal neurons of PS1M146V and 3xTgAD mice reduces ryanodine receptor (RyR)-mediated Ca^2+^ release, restores cAMP response element-binding (CREB)-dependent gene expression, rescues long-term potentiation (LTP) to normal levels, attenuates Aβ accumulation and pTau and reverses memory deficits [61]. Another study also showed a physical interaction of IP_3_R with PS1 (M146L) and PS2 (N141I) mutants stimulating IP_3_R gating activity [62] (Figure 2 and Table 1). Of note, our laboratory and others reported a major role of enhanced RyR expression and activity in AD [63,64,65,66,67,68]. Nevertheless, dedicated studies are still needed to demonstrate if the interaction between IP_3_R and FAD PSs mutants occurs preferentially in MAMs and to unravel the physiopathological contribution of RyRs in MAMs dysfunction related to AD.

### 2.3. PACS2-PSS1

Phosphofurin acidic cluster sorting protein 2 (PACS2) is a multifunctional sorting protein that interacts with several cargo proteins and regulates their location in MAMs [69]. PACS2 controls apoptosis induction, through the translocation of Bim (Bcl-2-like protein 11) to lysosomes [70], or Bid (BH3 interacting domain) to mitochondria [71]. The precise localization of PACS2 in MAMs is still uncertain. However, it has been demonstrated that PACS2 controls ER-mitochondrial apposition through its tethering action and is also involved in Ca^2+^ transfer from ER to mitochondria through IP_3_R localization [71]. Accordingly, PACS2 deletion leads to decreased ER-mitochondria contacts and triggers mitochondria fragmentation [71] and apoptosis [72].

PSS1 (phosphatidylserine synthase-1) is synthesized and specifically located in MAMs, supporting the direct transfer of lipids between ER and mitochondria [73]. PACS2 and PSS1 are functionally linked, since the overexpression of PACS2 raises the levels of PSS1 in MAMs [71].

PACS2 and PSS1 regulations in AD were reported in a study by Hedskog et al. showing enhanced PACS2 and PSS1 protein levels in AD transgenic mice expressing Swe/LDN FAD mutations [54]. More precisely, PACS2 protein expression was enhanced in the hippocampus of 2 month-old aged mice before Aβ plaque formation, and in the cortex and the cerebellum at 6 months. Meanwhile, PSS1 protein expression was only enhanced in the cerebellum starting at 6 months. Importantly, the authors also revealed enhanced PSS1 and PACS2 protein levels in human SAD brains (Figure 2 and Table 1). Enhanced PSS1 and PACS2 protein expressions were not corroborated by an increase in PSS1 and PACS2 mRNAs levels, suggesting that the regulation of PSS1 and PACS2 expressions in AD occurs at the protein level only. The authors also reported unchanged phospholipid metabolism in both mice and AD brains, thus questioning the functional consequences related to enhanced PSS1 and PACS-2 protein expression. Nevertheless, ablation of PSS2 triggers degeneration of astrocytes and neuronal hippocampal primary cultures. In conclusion, this unique study unravels the potential contribution of an additional MAMs molecular component in AD [54].

### 2.4. BAP31-FIS1-DRP1

Another physical tether of MAMs has been described as the interaction between the resident integral ER protein B cell receptor–associated protein 31 (BAP31) and the OMM mitochondrial FIS1 protein involved in the initiation of mitochondrial fission process [74,75]. FIS1 interacts with dynamin-related protein1 (DRP1) after its recruitment at mitochondrial fission sites by the OMM receptors (Mff, Mid49 and Mid51) in the MAMs complex, including BAP31 [76]. Intriguingly, a recent study reported that the human FIS1 protein can cause mitochondrial fragmentation in the absence of DRP1 and Dyn2 GTPases through its ability to bind to MFN1, MFN2, and OPA1, also inhibiting the mitochondrial fusion [77]. Shen et al. also demonstrated that mutations in the *FIS1* gene do not cause defects in mitochondrial fission in response to fission-inductor chemical treatments but trigger persisting large aggregates of autophagosomes [76]. Accordingly, FIS1 KO MEFs cells display a strongly impaired mitophagy [78]. Iwasawa et al. showed that FIS1 conveys an apoptosis signal from the mitochondria to the ER through its interaction with BAP31 at the ER and its cleavage into the pro-apoptotic p20 [74]. The latter triggers a Ca^2+^ transfer from the ER to the mitochondria leading to ΔΨm depolarization, hence priming cell death induction [74,75]. BAP31 is also essential for mitochondrial homeostasis as it stimulates the formation of the mitochondrial complex I [79].

Several studies reported that FIS1, BAP31, and DRP1 may contribute individually to AD physiopathology (Figure 2 and Table 1). It was first reported that the mRNA and protein levels of FIS1 and DRP1 are increased in the frontal cortex of patients with early, definite, and severe AD [42]. Joshi et al., reported later that Aβ_42_-treated neurons and fibroblasts from SAD or FAD patients feature an increased interaction between DRP1 and FIS1, which causes mitochondrial dysfunctions attested by increased mitochondrial fragmentation, oxidative stress, decreased ΔΨm and ATP levels, and increases in the activity of pro-apoptotic enzymes and Cyt c release [80]. Most importantly, inhibiting the DRP1-FIS1 interaction corrects these mitochondrial impairments, reduces in the 5xFAD mice AD model (that overexpress human APP and PS1 transgenes with a total of five AD-linked mutations) cognitive defects, Aβ_40_ and Aβ_42_ levels, and oxidative stress, and increases ATP levels [80]. In parallel, BAP31 knockdown in the hippocampus and the cerebral cortex of PS1 M146V mice results in an increased BACE1 protein level, exacerbating C99 accumulation and Aβ plaque formation [81]. Other studies showed that pTau and Aβ monomers and oligomers can also directly interact with Drp1 in AD late stages, likely contributing to abnormal mitochondrial dynamics [42,82]. Moreover, Aβ also triggers nitric oxide production, likely contributing to Drp1 S-nitrosylation-mediated mitochondrial fragmentation and neuronal damage [83] (Figure 2 and Table 1). Together, these studies support the involvement of the BAP31-FIS1-DRP1 complex in AD pathology.

### 2.5. VAPB-PTPIP51

The OMM protein, tyrosine phosphatase-interacting protein-51 (PTPIP51), was identified as a binding partner for the resident ER protein vesicle-associated membrane protein-associated protein-B (VAPB) [84]. As a physical tether of MAMs, VAPB has several functions, including vesicle trafficking and UPR [84]. It was then demonstrated that the loss of either VAPB or PTPIP51 perturbs the uptake of Ca^2+^ by mitochondria, decreasing ATP production [85] and stimulating the formation of autophagosomes [86]. Additionally, PTPIP51 can interact with the oxysterol-binding protein (OSBP)-related proteins ORP5 and ORP8 in MAMs [87], likely contributing to phosphatidylserine (PS) transport between ER and mitochondria.

VAPB P56S mutation is associated with dominantly inherited familial forms of type-8 amyotrophic lateral sclerosis (ALS) [88]. Moreover, TAR DNA binding protein 43 (TDP-43) and fused in sarcoma (FUS), two ALS/frontotemporal dementia (FTD)-associated proteins, were reported to activate the glycogen synthase kinase-3β (GSK-3β), which inhibits VAPB-PTPIP51 binding, ultimately resulting in disrupted Ca^2+^ homeostasis and reduced ATP production [85,89]. Strikingly, it has recently been observed that AD patients with granulovacuolar degeneration bodies (GVB) feature lower VAPB level [90] (Figure 2 and Table 1). Interestingly, AD is a pathology where TDP-43 and FUS accumulate in stress granules and GVB. Furthermore, GSK-3β is also overactivated in AD [91]. A recent study reports a reduction of VAPB and PTPIP51 interaction in the pyramidal cortex of patients with early/mid dementia, and a reduction of the expression of both VAPB and PTPIP51 in the cortex of AD patients at late-stages [92]. Together, these studies also suggest the involvement of VAPB-PTPIP51 physical/or functional alterations in AD pathophysiology.

### 2.6. Sig-1R-Grp78

Sigma non-opioid intracellular 1-receptor 1 (Sig-1R) is a chaperone protein present in ER lipid rafts that interacts and modulates different proteins in ER and plasma membrane [93]. Sig-1R is essential for MAMs stability [94], and is implicated in lipid synthesis and trafficking [95].

Grp78 (or BiP), a member of the heat shock protein 70 family, is a chaperone and an ER stress sensor [96]. Importantly, Grp78 is present in MAMs, where it folds the steroidogenic acute regulatory protein (StAR) transporting cholesterol for delivery to the OMM [97]. Under ER stress conditions, Grp78 dissociates from and activates three branches of the UPR: activating transcription factor 6 (ATF6), IRE1α, and PERK. The UPR generates an adaptive or a pro-apoptotic response according to the stress duration and/or intensity [98]. In aging, the pro-apoptotic response becomes more common than the adaptive pathway, probably due to a failure in chaperone systems [98]. Interestingly, the impairment in UPR, apoptosis, and accumulation of misfolded proteins are common features of AD and other neurodegenerative diseases [99]. Desipte this, there is still no consensus on the role of Grp78 in AD. While APP/PS1 mice and Aβ_25-35_ treated neurons presented increased Grp78 expression (reviewed in Reference [100]), another study in AD post-mortem brains with PS1 mutations showed a reduction in Grp78 mRNA levels [101]. Recently, a study demonstrated that the 5xFAD mice had no changes in Grp78 expression and other ER stress-related proteins [102]. These discrepancies could be linked to the pleiotropic functions regulated by and regulating Grp78 expression (Figure 2 and Table 1).

Sig-1R forms a complex with Grp78 in MAMs under resting conditions [103]. During ER stress, Sig-1R also dissociates from Grp78 and forms a complex with IP_3_R through ankyrin B to increase Ca^2+^ signaling from ER to the mitochondria, thereby enhancing ATP synthesis [104]. Besides, Sig-1R stimulates phospholipase C (PLC) that increases IP_3_ levels, resulting also in Ca^2+^ release from the ER [105]. The activation of Sig-1R is considered neuroprotective since Sig-1R silencing in hippocampal neurons results in Cyt c release, caspase-3 activation, and a deficit in spine formation. Remarkably, this mechanism is reversed by superoxide dismutase activation, showing that the reduction of Sig-1R increases the levels of ROS [106].

Interestingly, Sig-1R has been associated with a variety of neurodegenerative diseases, including AD [107,108]. Reduced Sig-1R binding sites were reported in AD post-mortem brains [109]. It has also been proposed that a polymorphism in Sig-1R (TT-P) correlates with the risk of developing AD [110]. This finding has been challenged in another study demonstrating that another Sig-1R polymorphism (Q2P) may worsen clinical outcomes only in association with the gentic risk factor APOEε(epsilon)4 [111]. The protective effect of Sig-1R has also been observed in two AD mice models (3xTg-AD and MacGill-R-Thy1-APP), where the treatment with a Sig-1R agonist, named AF710B (a concomitant agonist of the muscarinic 1 receptor), have been shown to restore cognitive impairment, and to reduce amyloid pathology and neuroinflammation [112,113]. Accordingly, the silencing of Sig-1R or application of the Sig-1R antagonist (NE-100) aggravated the pathological status in Aβ_25-35_ treated mice (i.e., learning deficits, reduced BDNF levels, and enhanced expression of Bax) [114]. The treatment of Aβ_25-35_ injected mice with ANAVEX2-73, another agonist of Sig-1R, decreases pTau, Bax/Bcl-2 ratio, lipid peroxidation, C99 accumulation, and Cyt c release [115,116], while decreasing Sig-1R expression enhances Tau hyperphosphorylation and reduces dendritic spines formation [117].

Overall, these studies converge to demonstrate the neuroprotective effect of Sig-1R agonists, acting through a diversity of Sig-1R-mediated signaling functions that are generally pro-survival and anti-apoptotic.

### 2.7. Protein Kinase-Like Endoplasmic Reticulum Kinase: PERK

Several studies reported that the activation of the PERK pathway attenuates protein synthesis until the unfolded protein accumulation is removed [98]. Interestingly, PERK is enriched at MAMs [118], where it facilitates the tethering of the ER to mitochondria and sensitizes cells to apoptosis [118,119]. PERK KO cells show reduced MAMs and disturbed cytosolic Ca^2+^ signaling and also protect cells from ROS-induced mitochondrial dysfunction [118]. A recent study demonstrates that PERK localized in MAMs phosphorylates mitochondrial E3 ubiquitin ligases and decreases the formation of mitochondria-associated ER membranes under ER stress condition [120]. UPR occurs relatively early in AD, and PERK was shown to be activated in human AD-derived brains [121,122]. The dysregulated PERK pathway has been recapitulated in AD mice models that develop β-amyloidosis [123,124,125], Tau-mediated neurodegeneration [126,127], and in SAD genetic risk factor APOEε4 mice models [128] (Figure 2 and Table 1). Several studies have provided mechanistic insights into the direct relationship between PERK overactivation and several paradigms occurring in AD. The phosphorylation of eIF2α, occurring down-stream of PERK activation, has been shown to increase BACE1 expression, thereby enhancing the production of Aβ in neurons [129], and genetic reduction of PERK reduces BACE1 protein level and Aβ production in the 5xFAD mouse model [123]. Accordingly, the selective ablation of PERK improved the synaptic plasticity and spatial memory in mice harboring APP and PS1 mutations [124], consistent with the requirement for active protein translation in memory consolidation [130]. Another study demonstrated that a reduction in eIF2α phosphorylation enhances the late phase of LTP and memory in mice [131]. Furthermore, the local expression of ATF4 (a transcription factor activated downstream of PERK and eIF2α ER stress responsive pathway) in axons induces axonal damage through a cell-non-autonomous mechanism that propagates between neurons [132]. These data suggest that sustained PERK-eIF2α-ATF4 activation worsens AD pathogenesis, and that PERK localization in MAMs may act as a new pathogenic route contributing to AD development.

**Table 1:** MAMs molecular component alterations in AD. These alterations include reduced (↓), increased (↑), unchanged expressions (=), or interaction (⟷) between proteins observed in AD study models and human-derived samples (SAD brains and SAD/ FAD fibroblasts); KO: knock-out; KI: knock-in; “OE”: overexpressing; MCI: mild cognitive impairment; GVB: granulovacuolar degeneration bodies; Aβo: Aβ oligomers.

Proteins	Alterations	Study Models	References
MFNs	Polymorphism	AD patients	[40]
	↓MFN2 & MFN1 (proteins and mRNA)	AD brains (hippocampus & frontal cortex) & N2a “OE” APPswe	[41,42,43]
	↑MFN2	MCI cytoplasmic hybrid (cybrid) cells	[44]
	↑MFN2⟷PS2 (FAD), ↑MAMs	MFN2 KO MEFs FAD PS2 (N141I) Mice	[46]
IP_3_R-Grp75-VDAC	↑VDAC Aβ⟷VDAC pTau⟷VDAC	Tg2576 and J20 mice APP, APP/PS1, 3xTgAD mice & AD brains (cortical tissues)	[53] [55]
	↑VDAC, ↑IP_3_R activity, ↑MAMs	Aβ-treated hippocampal neurons	[54]
	VDAC dephosphorylation	AD brains	[56]
	↓Grp75	AD brains (temporal and parietal cortex)	[52]
	FAD PS1/2⟷IP_3_R	Sf9 cells	[62]
PACS2-PSS1	↑PACS2, ↑PSS1	APPs_we/LDN_ mice AD brains cortex	[54]
BAP31-FIS1-DRP1	↑Fis1, ↑Drp1	AD brains (frontal cortex)	[42]
	↑Drp1⟷Fis	Aβ_42_-treated neurons, SAD/FAD fibroblasts, N2a “OE” APPswe, & 5xTgAD mice	[80]
	pTau⟷Drp1 ↓Drp1 mito localization	AD brains, APP, APP/PS1 & 3xTgAD mice *Drosophila* “OE” Tau R406W	[55] [133]
	Aβ⟷Drp1	AD brains (frontal cortex)	[42]
VAPB-PTPIP51	↓VAPB, ↓PTPIP51 ↓VAPB⟷PTPIP51	AD brains (subicular with GVB neurons) AD brains (cortex)	[90] [92]
Sig-1R-Grp78	↓Sig-1R Sig-1R polymorphism	AD brains AD patients	[109] [110,111]
	↑Grp78	Streptozotocin model, APP/PS1 mice & Aβ_25-35_-treated rats	[134,135,136]
	↓Grp78 mRNA	AD brains (FAD PS1)	[101]
	=Grp78 expression	5xFAD mice	[102]
PERK	↑PERK pathway	AD brains 5xFAD mice APP/PS1, APP(SL)/PS1 KI & rTg4510 mice APOE4 mice	[121,122,123,124,125,126] [123] [124,125,126,127] [128,129]
IRE1α	↑↓IRE1α pathway, ↑↓XBP1s	AD brains & 5xFAD mice Aβ_42_o-treated SH-SY5Y & CHO “OE” APP_LDN_	[137] [138]
S1T	↑S1T	AD brains, SHSY-5Y “OE”APPswe or treated with Aβo	[139]

### 2.8. Active Inositol-Requiring Transmembrane Kinase and Endonuclease Alpha: IRE1α

The UPR IRE1α branch increases protein folding trough the induction of chaperones and enhances the removal of misfolded proteins [98]. Active IRE1α processes the mRNA encoding X-box-binding protein 1 spliced isoform (XBP1s), a transcription factor that upregulates genes encoding mediators of protein folding, ERAD (ER-associated degradation), organelle biogenesis, and protein quality control [98]. A fraction of IRE1α is located at MAMs through Sig-1R stabilization responding to mitochondrial ROS [103,140]. A recent study demonstrated that the mitochondrial ubiquitin ligase (MITOL/MARCH5) inhibits ER stress-induced apoptosis through ubiquitylation and degradation of IRE1α at MAMs [141]. IRE1α at MAMs can also induce cell death via mitochondrial Ca^2+^ overload [142]. Another study linked IRE1α to mitochondrial dysfunction and apoptosis through the phosphorylation of ASK1 and JNK [143,144].

The activation of the IRE1α branch is implicated in AD pathogenesis, mainly through the activation of XBP1s [121,122] (Figure 2 and Table 1). Enhanced or reduced XBP1 mRNA splicing was reported in AD human brains [145]. XBP1s activation occurs upon Aβo treatment lowering BACE1 protein level [138] and activating Kalirin-7 (Kal7), a protein that controls synaptic plasticity [146]. This suggest that XBP1s acts as a protective mechanism in AD. Accordingly, the overexpression of XBP1s protects against Aβ toxicity [147] and pathological Tau [148]. In addition, a genomic screen identified a cluster of AD genes as possible direct targets of XBP1s, including APP, and components of the α-secretase, and proteins involved in APP trafficking and maturation [149]. Finally, in both *Drosophila* and mammalian cell culture models of AD, XBP1 overexpression also down-regulates RyR3 expression, which in turn prevents cytosolic Ca^2+^ overload [147]. These last data corroborate studies demonstrating that pharmacological and genetic approaches reducing RyR-mediated exacerbated ER Ca^2+^ leak provide protective effects and rescue several AD pathogenic paradigms in cellular and mice AD models [64,65,66].

### 2.9. The Truncated Variant of the Sarco-Endoplasmic Reticulum Ca^2+^-ATPase 1: S1T

S1T is among molecular partners at the ER-mitochondria interface regulating inter-organelle Ca^2+^ signaling and cell death [150,151,152]. We reported the induction of S1T expression under pathological ER stress conditions occurring through the PERK-eIF2α-ATF4-CHOP (C/EBP homologous protein) pathway. On the one hand, S1T induction amplifies ER stress response, and on the other hand, it enhances ER Ca^2+^ leak, increases the number of ER-mitochondria contact sites, and inhibits mitochondrial movements. This leads to increased Ca^2+^ transfer to mitochondria, thus activating the mitochondrial apoptotic pathway [152]. Interestingly, we recently reported a molecular interplay between S1T-dependent ER Ca^2+^ leak, UPR, and APP processing, likely contributing to AD pathogenesis [139] (Figure 2 and Table 1). We demonstrated that S1T expression is increased in SAD brains and correlates with Aβ and ER stress chaperone protein levels (Grp78 and Calreticulin). Increased S1T expression is induced in an Aβ-dependent manner and enhances in return the production of APP-CTFs and Aβ through specific increases of BACE1 expression and activity. Interestingly, the induction of S1T expression is also linked to neuroinflammation [139]. Other studies are ongoing to unravel the contribution of S1T-mediated ER Ca^2+^ leak to MAMs dysfunctions, synaptic plasticity alterations, and cognitive deficits in AD.

## 3. The MAMs Hypothesis in the Pathophysiological Process of Alzheimer’s Disease

In this chapter, we focus on the role of main AD players in mitochondrial and ER dysfunctions and their link with MAMs alterations in AD.

### 3.1. Apolipoprotein E: APOE

APOE is a glycoprotein mostly expressed in astrocytes, microglia, and neurons under stress conditions, and in the choroid plexus in the CNS [153,154]. APOE plays a relevant role in lipid transport, in particular cholesterol and cholesterol esters. This occurs via APOE binding to a low-density protein (LDL) receptor, such as LDLR and LDLR-related protein 1 (LDLR-1) [155]. APOE is essential for brain functions since APOE KO mice present synaptic loss and cognition dysfunction. The rescue of APOE in the periphery can improve learning and memory impairments, but without affecting synaptic loss [156]. APOE is a highly polymorphic gene encoding for three major APOE epsilon (ε) isoforms (APOEε2, APOEε3, and APOEε4). As stated above, APOE is a genetic risk factor for LOAD forms [3,4]. The elevated risk of AD correlates with the number of APOEε4 alleles [157]. While APOEε2 confers a decreased risk of AD in comparison with the APOEε3 allele, a single APOε4 allele increases the risk of AD four-fold compared to the more common APOEε3/APOEε3 genotype [157]. The presence of two APOEε4/APOEε4 alleles increases the risk of AD by approximately twelve-fold [158]. APOE polymorphism causes alterations in APOE structure and function that results in a different binding to lipids [157]. Notably, there is a relationship between APOE polymorphism and enhanced Aβ pathology [159].

Mitochondrial dynamics are differently impacted by APOE isoforms. APOEε*4* knock-in (KI) mice exhibited higher expression of MFN1 and lower expression of DRP1 in the hippocampus in comparison to APOEε3-expressing mice. The decrease in DRP1 was further confirmed in AD patients carrying two APOEε*4* alleles [160]. The expression of COX1 and translocase of outer mitochondrial membrane 40 homolog (TOMM40) were also higher in APOEε4-expressing mice, which supports the idea of increased mitochondrial fusion [160]. The APOEε4 isoform is also associated with altered mitophagy, characterized by reduced expression of cleaved PINK1 and higher levels of Parkin and SQSTM1/p62 in the hippocampus of APOEε4-expressing mice. This latter presented elongated mitochondria with a smaller cristae density compared to APOEε3-expressing mice hippocampus, demonstrating the contribution of APOEε4 to mitochondrial structure alteration [160]. In addition, a proteomic study showed that N2a cells expressing APOEε4 had impaired respiration and increased glycolysis in basal conditions and presented less reserve capacity to generate ATP, decreased NAD+/NADH ratio, and increased ROS and the mitochondrial Ca^2+^ load under a stress condition in comparison to APOEε3-expressing cells [161]. Importantly, APOEε4-expressing cells also manifest increased levels of VDAC1, IP_3_R, BAP31, FIS1, and MFN2 and a decrease in Grp75 expression, showing that APOEε4 modulates several molecular components of MAMs [161]. As shown in Figure 1, one main function of MAMs is phospholipid synthesis, specially phosphatidylserine (PS), which is transported from the ER to mitochondria, where it is decarboxylated and transformed in phosphatidylethanolamine (PE) that is transported to the ER [162]. Human fibroblasts and mice hippocampal neurons subjected to conditioned media (CM) obtained from astrocytes isolated from APOEε3 and APOEε4 KI mice showed an increase in PS and PE that was larger with APOEε4 CM than with APOEε3 CM [163]. However, this difference was abolished in MFN2 KO MEFs, suggesting that APOEε4-mediated phospholipids synthesis is strictly dependent on the increased interaction between ER and mitochondria [163]. The same study also reported that APOEε4 CM enhances cholesterol oleate level compared to APOEε3 CM and that this effect is abolished in MFN2 KO MEFs. Accordingly, fibroblasts treated with APOEε4 CM presented increased cholesteryl ester synthesis and lipid droplets as compared to APOEε3 CM treated fibroblasts [163]. This study was the first to unravel a relevant role of the APOEε4 allele in controlling MAMs activity, likely contributing to LOAD development.

### 3.2. Presenilins

Both PS1 and PS2 are localized in the ER, Golgi, and golgian network, the plasma membrane, endosomes, lysosomes, nuclear membrane, and mitochondria [164]. Being catalytic subunits of the γ-secretase complexe, PSs are well known to be involved in APP metabolism, leading to the production of different fragments and defining the length of Aβ peptide. Currently, more than 320 mutations of PS1 and 63 mutations of PS2 have been shown to be involved in AD or other dementias (Alzforum: https://www.alzforum.org/mutations). Many of these mutations are responsible for the increased Aβ_42_/Aβ_40_ ratio. Of note, Aβ_42_ peptides are more toxic and prone to aggregation and accumulation in plaques than Aβ_40_ peptides. In addition, PSs participate in several functions closely linked to MAMs. First, it was described that PSs participate in autophagy by favoring the fusion of autophagosomes with lysosomes [164]. Neely et al. reported enhanced autophagosomes and LC3-II levels in fibroblasts isolated from PS1 or PS2 KO mice arguing for a defective lysosomal degradation of the autophagosome [165]. Moreover, Száraz and colleagues suggested that the alteration in autophagy involves Ca^2+^ homeostasis dysregulation associated with ER stress response, particularly to Grp78 enrichment in MAMs [166]. Interestingly, PSs are also involved in ER Ca^2+^ regulation [167], forming a passive ER Ca²^+^ leak channel [168], directly interacting with SERCA2b [169], or directly or indirectly impacting IP_3_R- and RyR-mediated ER Ca^2+^ release [68,170]. These data support the idea that FAD PSs dysregulate both ER Ca²^+^ store filling and emptying.

More recently, PS1 and PS2 have been reported to be enriched in MAMs [171], with PS2 being directly involved in the interaction between ER and mitochondria [46,172]. Filadi and colleagues showed that PS2 can directly interact with and requires MFN2 expression to modulate the coupling between ER and mitochondria [46]. The authors also showed that two familial mutants of PS2 (T122R and N141I), known to be involved in Ca^2+^ release during atypical dementia and Aβ_42_ production during AD, are more efficient in ER-mitochondria tethering than wild type (WT) PS2, increasing mitochondrial Ca^2+^ uptake upon ER Ca^2+^ release [46,173]. Furthermore, SH-SY5Y neuroblastoma cells expressing these PS2 mutants and FAD-PS2 patient-derived fibroblasts showed a blockade in the late phase of autophagy [174], as demonstrated by an increase of LC3-II and SQSTM1/p62 levels, and alteration of the RAB7-dependent autophagosome-lysosome fusion step as a consequence of the alteration of the Ca^2+^ content of intracellular stores and cytosolic Ca^2+^ signaling [174]. These studies demonstrate that the reinforcement of MAMs linked to FAD-PS2 mutants contributes to several mitochondrial dysfunctions reported in AD, in particular the disruption of subcellular Ca^2+^ handling and the regulation of autophagy [46,173,174].

Although FAD PS1 mutants do not seem to be directly involved in the ER-mitochondria tethering, several data show that PS1 FAD mutations alter several functions linked to MAMs. Firstly, several FAD PS1 mutants (M146V, L166P, A246E, E273A, G384A, and P436Q) were shown to exert a dominant-negative effect on ER Ca²^+^ leak and/ or to exacerbate IP_3_R- and RyR-mediated ER Ca^2+^ release and alter neuronal Ca²^+^ signaling [67,68,168,170,175]. Moreover, the expression of PS1-E280A FAD mutation in Purkinje cells and SH-SY5Y cells was shown to impair Ca²^+^ homeostasis associated with reduced levels of CACNA1A, IP_3_R1, and IP_3_R3, leading to decreased levels of Ca²^+^-dependent mitochondrial transport proteins (i.e., MIRO1 and KIF5C), and enhanced tethering of ER and mitochondria [176]. Finally, PS1-FAD fibroblasts (A246E, M233T, H163Y, M146L and L392V) were also described to impact autophagy by impairing autolysosome acidification and cathepsin D activation that are caused by the failure of PS1 to target the v-ATPase V0a1 subunit to lysosomes [177]. Together, these results suggest that PS1 is likely directly or indirectly involved in MAMs structure and functions.

To conclude, these data support the hypothesis that different pathways regulated by PSs converge towards MAMs-linked Ca²^+^ homeostasis, autophagy, and mitochondria function, the alterations of which represent common functional defects in AD.

### 3.3. APP-Derived C-Terminal Fragments (APP-CTFs) and Aβ

APP participates neuronal migration, neurites outgrowth, synaptogenesis, synapses stabilization, and synaptic plasticity [178]. As stated in the introduction, APP is synthesized in the ER and transits through the Golgi apparatus, where it undergoes several post-translational modifications such as N- and O-glycosylations, sialylations, phosphorylations, and sulfations before arriving at the plasma membrane in its mature form [178]. Several studies suggest that APP is also targeted to mitochondria [179,180] (Figure 3 and Table 2). Importantly, it was demonstrated that full-length APP interacts with the mitochondrial import machinery formed by TOMM40 and TIMM23, inhibiting Cyt c oxidase activity and increasing mitochondrial ROS and dysfunction [181]. This finding was further supported by the presence of APP catabolites (i.e., Aβ, C99, and AICD) in the mitochondria [182,183,184,185,186,187]. Supporting APP localization and its processing in MAMs, we and others demonstrated the presence of APP-CTFs and Aβ in MAMs and also of different components of the γ-secretase complex in ER and mitochondria [183,187,188,189,190]. Consistently, we also showed in vitro activities of both β- and γ- secretases in MAMs of AD mice brains [187]. It has been proposed that MAMs behave as a detergent-resistant lipid raft-like domain [190], sustaining β- and γ-secretase the activities in MAMs [191,192,193].

Most importly, we and others also evidenced an increase of ER and mitochondria contact sites in SH-SY5Y cells overexpressing APPswe [187], in fibroblasts from SAD and FAD individuals, as well as in MEFs isolated from mice simple KO for either PS1 or PS2 or both [190]. Increased ER and mitochondria tethering is consistent with the hypothesis that Aβ is produced at the interface between ER and mitochondria [194] and its subsequent transport into mitochondria via the translocase of the OMM (TOMM) machinery [194], trigerring mitochondria dysfunctions (i.e., enhanced ROS, reduced mitochondrial respiratory chain complex I activity, and interaction with several mitochondrial proteins) [195,196,197,198] (Figure 3). In addition, Aβ was shown to regulate fatty-acid metabolism, another function of MAMs, as revealed by neutral lipids accumulation found in neuroblastoma and CHO cells expressing APPswe or APP_LDN_ mutations [187] and in human-derived SAD and FAD fibroblasts [190]. While the toxicity of Aβ towards mitochondria is undoubtful, Pera et al. also demonstrated the toxic charge of C99 fragment in MAMs. Indeed, the authors showed in PSs double KO MEFs treated with DAPT (a γ-secretase inhibitor), an accumulation of C99 peptide triggering MAMs deregulation, as manifested by an upregulation of sphingolipid metabolism translated by a higher ceramide and sphingomyelin synthesis in mitochondrial membranes [186,199]. Interestingly, ceramide has been shown to alter mitochondrial lipid composition, disrupting ΔΨm and mitochondrial membrane permeability [200]. It was also suggested that mitochondrial ceramide elevation could disrupt the assembly and the activity of mitochondrial respiratory complexes and provoke mitochondrial dysfunctions and apoptosis [186]. Another study confirmed the contribution of C99 to lipid metabolism [201]. Hence, pathogenic accumulation of C99 in the ER induces excessive uptake of extracellular cholesterol, trafficking from the plasma membrane to the ER, resulting in the continuous formation, activation, and turnover of MAMs in AD [201] (Table 2). In a recent study, we demonstrated that APP-CTFs accumulates in mitochondria in cellular AD models (SH-SY5Y cells expressing either APPswe, or C99 fragment) as well as in brains of 3xTg-AD mice treated with γ-secretase inhibitor, and in C99-expressing mice [202]. We also demonstrated that APP-CTFs, independently from Aβ, triggers excessive mitochondrial cristae disorganization, enhances mitochondrial ROS production, and elicits basal mitophagy failure associated with deficient fusion of mitochondria with lysosomes. Importantly, we reported a correlation between APP-CTFs accumulation in mitochondria and the mitophagy failure molecular signature in human post-mortem SAD brains [202] (Table 2).

Taken together, these results led us to assume that APP-CTFs (particularly C99) is directly generated in MAMs, or targeted through an unknown mechanism to MAMs after being produced in endosomes. The overaccumulation of APP-CTFs in MAMs consequently triggers mitochondrial and lipid dysfunctions, and a mitophagy failure phenotype contributing to neuronal demise in AD.

### 3.4. Tau

Tau is an associated-microtubule protein contributing to axonal transport and synaptic plasticity [203]. Tau is involved in tauopathies such as FTD, fronto-temporal lobar degeneration with Pick bodies, corticobasal degeneration, and AD [204]. During the pathophysiological process of AD, Tau is abnormally hyperphosphorylated and loses its affinity for microtubules. pTau is then redistributed in the somato-dendritic compartment, where it aggregates in insoluble filaments forming NFTs and leading to neuronal dysfunctions through axonal transport alteration [203,205].

Even if there is no description of FAD linked to Tau mutations, “artificial” Tau mutations are used in fundamental research to yield Tau hyperphosphorylation and to mimic, at least in part, some aspects of AD-related Tau pathology. Interestingly, P301L mutation of Tau (Tau P301L), which is known to cause Tau hyperphosphorylation, was reported to decrease mitochondrial complex V levels, leading to impaired mitochondrial respiration and ATP synthesis as well as increased ROS production [206]. The same mutation in the N2a cell line was also shown to interact with Parkin and sequester it in the cytosol, triggering impaired mitophagy [207]. Besides, Tau overexpression in HEK293 cells and rat primary hippocampal neurons enhances the fusion of mitochondria by increasing the expression of fusion proteins OPA1, MFN1, and MFN2 triggering an alteration of mitochondrial dynamics and functions illustrated by decreased ATP level, ATP/ADP ratio, and complex I activity [208]. Perrault et al. revealed that Tau P301L increases the number of ER and mitochondria contacts in the spinal cord motor neurons. They also notice enhanced colocalization of Tau P301L with ER membranes in this model and also in AD brains [209], likely increasing ER-mitochondria tethering. In another study, Tau overexpression in CHO or N2a cells was reported to alter the distribution of mitochondria and ER to the cell periphery [210]. Afreen et al. then reported that calpain-mediated cleavage of Tau generating Tau_45-230_ fragment (occurring downstream of increased Ca^2+^ influx after exposure of hippocampal neurons to Aβo) is associated with the cytoskeleton plus membrane-bound organelles and reduces the number of mitochondria transported along hippocampal axons [211].

Together, these data suggest that the hyperphosphorylation and elevated intraneuronal concentration of Tau would affect the transport of mitochondria or ER dynamics, thus impairing the formation of ER and mitochondria contact sites in AD.

**Table 2:** Alterations of mitochondria- and MAMs-structure and functions associated with AD-related proteins. These alterations include: ↓ reduced or ↑ increased expression/activity, ⟷ interaction observed in AD study models (cells and mice brains) and human derived samples (SAD brains and SAD/FAD fibroblasts). Mito: mitochondria, KO: knock-out; PSDKO: PS1 and PS2 double KO; KI: knock-in; “SI”: silencing; “OE”: overexpressing; PS: phosphatidylserine; CO: cholesterol oleate; CE: cholesterol ester; CM: conditioned media, WT: wild type; CGC: cerebellar granule cells; ROS: reactive oxygen species.

Proteins	Alterations	Study Models	Ref
APOEε4	↓DRP1, ↑MFN1 Mito fusion (↑COX1, ↑TOMM40) Mitophagy failure (↓PINK1, ↑Parkin, ↑p62)	AD brains APOEε4 KI mice	[160]
	↑VDAC1, ↑IP_3_R, ↑BAP31, ↑Fis1, ↑MFN2, ↓Grp75	N2acells “OE” APOE4	[161]
	↑PS, ↑CO, ↑CE, ↑Lipid droplets	APOE4 KI mice astrocytes CM treatment	[163]
PS1 & PS2	↑LC3-II and ↑autophagosomes	MEFs (PS1KO, PS2KO, & PSDKO) HepG2 cells (PS1“SI”)	[165,166]
	PS2⟷Mfn2 ↑MAMs	MEFs (MFN2 KO), Human FAD fibroblasts, SHSY-5Y & primary neurons “OE” MFN2	[46,172]
	↑ER-mitochondria coupling Autophagy blockade (↑LC3-II, ↑p62)	FAD PS2 (T122R, N141I) &. SHSY-5Y “OE” FAD PS2	[174]
	↓IP_3_R1/3, ↓MIRO1, ↓KIF5C ↑MAMs	Purkinje & SHSY-5Y “OE” FAD PS1(E280A)	[176]
	Autophagy impairment	FAD PS1 (A246E, M233T, H163Y, M146L, L392V) fibroblasts	[177]
	↑MAMs ↑ MAMs function (↑CE, ↑PS, ↑PE, ↑lipid droplets)	PS1 KO, PS2 KO, PSDKO FAD and SAD patient fibroblasts	[190]
APP, Aβ & APP-CTFs	APP in mitochondria	HCN-1A, COS cells “OE” APPWT or APP chimeric mutants & APPswe mice	[169,180,181]
	APP, APP-CTFs, Aβ in MAMs ↑MAMs ↑ceramide, ↓sphingomyelin,↑lipid droplets (Aβ dependant)	SHSY-5Y “OE” APPswe, APP_LDN_ Mice brains (WT,APP23 & APP23/PS45) PSDKO +/− DAPT	[185,186,187,190,194]
	↑mito ROS, ↓complex I &IV activities (Aβ-dependant)	AD brains; Aβ-treated CGCs	[195,196]
	↑mito ROS, ↓complex I activity Mitophagy failure (APP-CTFs dependant)	3xTgAD, 2xTgAD, AAV-C99 mice, SHSY-5Y cells “OE”APPswe or C99 +/− γ-secretase inhibitor AD brains	[202]
Tau	↓complex V, ↓ATP, ↑mitROS	Tau_P301L_ mice	[206]
	Mitophagy failure (↓Parkin mitochondrial translocation)	N2a “OE” Tau_P301L_	[207]
	↑OPA1, ↑MFN1/2, Mitochondrial fragmentation, ↓complex I activity, ↓ATP	HEK293 & rat primary hippocampal neurons “OE” Tau	[208]
	Mitochondria & ER distributions	CHO and N2a cells “OE” Tau	[210]
	↓Mito axonal movement	Hippocampal neurons “OE” Tau45-230	[211]
	↑MAMs	Motor neurons “OE” Tau_P301L_	[209]
	↓MAMs	Tau_P301L_ mice	[212]

## 4. MAMs as a Potential Therapeutic Strategy

The studies highlited above tend to demonstrate that MAMs structure and function alterations could play a role during the pathophysiological process of AD. Taking this into account, we may assume that MAMs should constitute a new therapeutic target in AD. Up to now, different assays were driven to target either ER or mitochondrial dysfunctions in neurodegenerative diseases, as an indirect way to alleviate MAMs-associated dysfunctions. Among them, Salubrinal, a selective inhibitor eIF2α phosphatase enzymes, prevents ceramide-induced apoptotic cell death through the inhibition of IRE1α /apoptosis signal regulating kinase 1 (ASK1)/c-Jun N-terminal kinase (JNK) phosphorylation [213] and decreases apoptosis in neurons and microglia exposed to Aβ [214,215]. Salubrinal also maintains eIF2α phosphorylated, thus inhibiting the synthesis of ER secretory proteins and decreasing proteoglycans and other profibrotic proteins, then facilitating axonal regeneration [216]. Celastrol and Tanespimycin, which target the heat shock protein response, are other potential drugs showing promising results in transgenic mouse models of AD by preventing NFκB activation, inhibiting BACE1 expression and reducing Aβ_40_, Aβ_42_ levels as well as C99 and sAPPβ production and NFTs [217,218].

Among mitochondrial targets, mitochondrial division inhibitor-1 (mdivi-1), a selective inhibitor of mitochondrial fission through the targeting of DRP1, has been reported to display neuroprotective properties by rescuing propofol-induced toxicity in stem cell-derived neurons [219]. In microglial cells, inhibition of mitochondrial fission by mdivi-1 significantly reduced Aβ-induced expression of CD11b (a pan-macrophage marker) and reversed cell death. Importantly, mdivi-1 treatment also markedly reversed mitochondrial dysfunction (loss of ΔΨm and Cyt c release) and caspase-3 activation) [220]. These results were further confirmed in neuroblastoma N2a cells subjected to Aβ_42_ treatment, showing that mdivi-1 enhanced mitochondrial fusion activity, lowered fission machinery, and increased biogenesis and synaptic proteins. These features were associated with elevated cell viability in cells exposed to Aβ and treated with mdivi-1 [221]. The same group reported synergistic protective effects of a mitochondria-targeted antioxidant SS31 and mdivi-1 in N2a cells expressing APPswe/LDN mutations by increasing mitochondrial DNA copy number and cell survival, reducing mitochondrial dysfunctions. Altogether, these data suggest that mdivi-1 exerts neuroprotective effects against Aβ-induced mitochondrial structure and function alterations and apoptosis [222]. Another mitochondrial fission inhibitor, P110, abrogating the interaction between DRP1 and FIS1, was shown to inhibit excessive fission as well as to restore mitochondrial function [223]; however, its therapeutic potential is still not demonstrated in the context of AD.

Few studies pointed to MAMs as a potential therapeutic target. Arismendi-Morillo et al. showed that restriction of glucose or ketogenesis influences the structure and function of MAMs and could be used as a therapeutic approach in astrocytic tumors [224]. Lynes et al. also found that palmitoylation of cysteine residues could assemble ER membrane proteins in MAMs [225]. The use of artificial linkers to control the connections between ER and mitochondria was also proposed. The expression of an ER-mitochondria linker in a *Drosophila* model of AD increases physical coupling between both organelles, raises Ca²^+^ levels in mitochondria known to be detrimental to their function. Indeed, authors also noticed an increase of ROS production in flies expressing the linker. However, while the expression of ER-mitochondria linker in flies reverses electron transport and extends *Drosophila* lifespan, authors report negative outcomes in mice [226]. Rapamycin-inducible artificial linkers with different lengths [227] allow to demonstrate that distinct processes may require an optimal spatial ER-mitochondria distance. Indeed, while a loose connection is needed for Ca²^+^ delivery, tighter connections are required for lipid and protein transfer between organelles [227]. A recent study proposed urolithin A (produced in the gut microbiome) as a pharmacological tool to control ER-mitochondria contacts and Ca^2+^ homeostasis in AD [228]. Urolithin A lowered mitochondrial Ca^2+^ influx and significantly alleviated high glucose-induced mitochondrial ROS accumulation. Most importantly urolithin A reduces the expression of APP, BACE1, as well as Aβ production and Tau phosphorylation [228].

## 5. Conclusions

A large number of studies delineated the proteome composition of MAMs and deciphered the molecular and functional links between MAMs dysfunctions and AD, where disturbances of mitochondrial and ER Ca^2+^ signaling were consistently reported. Besides Ca^2+^ tunneling and apoptosis, MAMs are also the hub for autophagosome and mitophagosome membranes biosynthesis and lipid biosynthesis that are all cellular hallmarks that were reported to be perturbed in AD study models and most importantly in human AD-derived samples. About 30 million individuals are estimated to be affected by AD worldwide and to date, no effective treatment exists to arrest disease progression. This review highlights the relevance of MAMs dysfunctions in AD development and pinpoints the possibility to target MAMs molecular components as therapeutical targets to treat or alleviate or slow down AD pathogenesis.
